# First detection of V410L *kdr* mutation in *Aedes aegypti* populations of Argentina supported by toxicological evidence

**DOI:** 10.1186/s13071-024-06405-3

**Published:** 2024-08-06

**Authors:** Paula V. Gonzalez, Aline C. Loureiro, Andrea Gómez-Bravo, Paola Castillo, Manuel Espinosa, José F. Gil, Ademir J. Martins, Laura V. Harburguer

**Affiliations:** 1 Centro de Investigaciones de Plagas e Insecticidas (CIPEIN-UNIDEF/CITEDEF/CONICET), Juan B. de La Salle 4397, Villa Martelli, B1603ALO Buenos Aires, Argentina; 2 Laboratório de Biologia, Controle e Vigilância de Insetos Vetores, Instituto Oswaldo Cruz, Fundacão Oswaldo Cruz (FIOCRUZ), Manguinhos, Rio de Janeiro, Brazil; 3 Fundación Mundo Sano, Ciudad Autonoma de Buenos Aires (CABA), Buenos Aires, Argentina; 4 Instituto de Investigaciones en Energía no Convencional, Grupo de Ambiente y Salud, Universidad Nacional de Salta, Orán, Argentina

## Abstract

**Background:**

*Aedes aegypti* (L.) is the main vector of dengue, yellow fever, Zika, and chikungunya viruses in many parts of the world, impacting millions of people worldwide each year. Insecticide-based interventions have been effective in controlling *Aedes* mosquito populations for several years, but in recent times, resistance to these compounds has developed, posing a global threat to the control of this mosquito.

**Methods:**

Ovitraps were used to collect *A. aegypti* eggs in the cities of Tartagal and San Ramón de la Nueva Orán (Salta), Puerto Iguazú (Misiones), and Clorinda (Formosa). World Health Organization (WHO)-impregnated papers with the discriminating concentration (DC) of permethrin, 5X, 10X and pirimiphos methyl were used for the toxicological bioassays. We also genotyped each sample for the three *kdr* single nucleotide polymorphisms (SNP): V410L, V1016I, and F1534C in individual TaqMan quantitative PCR (qPCR) reactions.

**Results:**

All investigated *A. aegypti* populations were highly resistant to permethrin, as the mortality percentage with the permethrin 10×DC remained below 98%. However, all populations were 100% susceptible to pirimiphos-methyl. *Kdr* genotyping demonstrated the presence of the V410L mutation for the first time in Argentina in all the populations studied. A prevalence of the triple mutant genotype (LL + II + CC) was observed in the northeastern cities of Clorinda (83.3%) and Puerto Iguazú (55.6%).

**Conclusions:**

This study demonstrates for the first time the presence and intensity of resistance to permethrin in different populations from Argentina, and correlates the observed phenotype with the presence of *kdr* mutations (genotype).

**Graphical Abstract:**

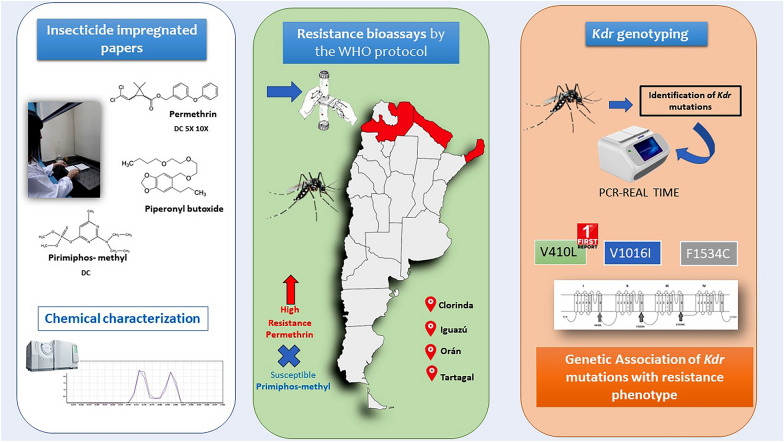

## Background

*Aedes aegypti* (L.) is the primary vector of dengue, yellow fever, Zika, and chikungunya viruses in many parts of the world, impacting millions of people globally each year. The most widely employed strategy to reduce *A. aegypti* densities is focused on the larval stages (removal of breeding sites, larvicide, and community education) to diminish the population of new adults. Furthermore, adult control using spatial sprays with adulticides is recommended during dengue outbreaks [1]. Insecticides such as the organophosphate temephos, which has very low toxicity to mammals, as well as insect growth regulators (such as pyriproxyfen and methoprene) and bacterial toxins (such as *Bti*), can be used in mosquito breeding sites to target mosquito larvae. Over the last 10 years, there has been a gradual increase in the use of pyrethroids, such as deltamethrin and permethrin, surpassing the proportional use of all other insecticides for controlling *A. aegypti* adults [2]. Insecticide-based interventions have been successful in efficiently controlling *Aedes* mosquito populations for many years, but because of intense use and reliance on the especially few active ingredients registered for public health applications, resistance to these compounds has been evolving and is currently a global threat to the control of this mosquito [3].

Resistance to all four classes of insecticides (carbamates, organochlorines, organophosphates, and pyrethroids) has developed in *A. aegypti* [3]. This resistance is increasingly undermining the effectiveness of control efforts. For instance, the resistance to pyrethroids is negatively impacting on adulticide campaigns in Caribbean [4, 5], while temephos resistance significantly reduced the duration of larviciding control in Cuba [6, 7].

Since 1997, the northwest and northeast regions of Argentina have experienced frequent dengue epidemic outbreaks [8]. Due to the significant growth in *Aedes* mosquito populations [9] and in response to the increased frequency of outbreaks and epidemics, there has been a significant rise in insecticide-based vector control interventions. These interventions involve the use of the organophosphate temephos for larvicidal treatment in water containers, and the pyrethroid permethrin as an adulticidal ultra-low volume (ULV) formulation.

Susceptibility levels to insecticides used on *A. aegypti* must be continually monitored in different geographical areas to develop effective control strategies [10]. The knockdown effect, or rapid paralysis, is a key feature of pyrethroid action on insects. This occurs due to the prolonged activation of voltage-gated sodium channels (NaV) by pyrethroids, ultimately leading to the blockage of action potential conduction [11]. Alterations in the *Na*_*V*_ gene associated with resistance to the knockdown effect are therefore referred to as knockdown resistance mutations (*kdr*), one of the major mechanisms of resistance to pyrethroids [12]. In Argentina, there were no variations in the susceptibility status data for pyrethroids during monitoring evaluations until 2013. Control failures were detected in Salvador Mazza (Salta, Argentina) at that time. The first publication about insecticide resistance of adult *A. aegypti* in Argentina reported a resistance ratio 50 (RR_50_) of 10.3 for *cis*-permethrin, which is considered a high resistance level. The results revealed resistance to deltamethrin (22.6% mortality using DC) and permethrin (53.6% mortality). However, complete susceptibility to the organophosphate malathion (100% mortality) was observed [13].

Several *kdr* mutations have been documented in the sodium channels of *A. aegypti* populations [14–16]. However, only some of these mutations reduce the sensitivity of the sodium channels to pyrethroids [11, 17, 18]. The simultaneous presence of resistance-associated mutations is frequent in pyrethroid-resistant *A. aegypti* populations, presumably resulting in elevated levels of resistance. In South and North America, the mutation V1016I often coexists with F1534C [19–23]. Vera-Maloof et al. [24] proposed a hypothesis of sequential evolution of these two *kdr* mutations. They predicted that the F1534C mutation was selected first and confers a low level of pyrethroid resistance, and that the V1016I haplotype likely has a fitness cost and cannot be selected in the absence of F1534C. The V1016I then arose from the F1534C haplotype and was rapidly selected because the double mutations confer a higher level of pyrethroid resistance. The mutation known as V410L was first reported in the sodium channels of mosquitoes by Haddi et al. [25], demonstrating that this valine to leucine substitution reduced the sensitivity of these channels to both type I (i.e., permethrin) and type II pyrethroids (i.e., deltamethrin). The presence of both V410L and F1534C mutations may account for the extremely high levels of resistance observed in the study. This suggests that monitoring strategies should prioritize screening for more than one sodium channel region. Implementing a strategy of alternating between type I and type II pyrethroid products for control could be effective, as the F1534C mutation has been shown to decrease the sensitivity of *A. aegypti* Nav1-1 channels to type I pyrethroids but not type II pyrethroids [15, 18]. However, the significant levels of pyrethroid insensitivity resulting from the V410L mutation alone or in combination with F1534C could render pyrethroids not only largely ineffective in regions where *A. aegypti* carry these combined mutations, but also exacerbate the situation by increasing the selection pressure for resistant individuals [25].

The *kdr* mutations V1016I and F1534C were reported in Argentina for the first time in *A. aegypti* from the city of Posadas (Misiones), in northeastern Argentina [26]. These same *kdr* mutations were also observed in *A. aegypti* populations of the northern provinces of Salta and Jujuy, and the Buenos Aires Metropolitan Area [27]. In the northern region, there were higher frequencies of alleles associated with resistance. However, like the work by Fay et al. [26], no bioassays were performed to show that the genotype found corresponds to populations phenotypically resistant to pyrethroids, especially considering that the frequency of the genotype that confers high resistance (II/CC) was lower than 15%.

These records highlight that *kdr* is starting to spread in *A. aegypti* populations from Argentina, potentially reducing the efficacy of current insecticide-based control strategies. However, there is a lack of accessible data documenting the prevalence and mechanisms of insecticide resistance at specific geographic allocations in this country. Such data are crucial for guiding national programs in selecting the most effective compounds for each resistance scenario.

In this work, we studied pyrethroid resistance in *A. aegypti* from four locations of Argentina with high selection pressure with insecticides in the northwest of the country. Furthermore, the presence of the V410L *kdr* mutation was detected for the first time in Argentina. The implications of this finding, along with the intensity of the resistance, the mechanisms involved, and control alternatives for the populations that were resistant, are discussed.

## Methods

### Chemicals

Technical grade permethrin 96% (permethrin cis: trans ratio of 45:55) was supplied by Chemotecnica S.A., Argentina. Pirimiphos-methyl (97.7%) and piperonyl butoxide (PBO) were purchased from Sigma-Aldrich S.R.L. Argentina. Silicone oil Dow Corning 556 was purchased from Daltosur S.R.L., Buenos Aires, Argentina. All solvents used (acetone) were analytical grade.

### Biological material

An insecticide-susceptible strain Rockefeller of *A. aegypti* was used. It originated from a strain in Venezuela and was reared since 1996 in our insectary at 25 ± 2 °C, 60–70% RH, and had a photoperiod of 12:12 h (L:D), with no exposure to pathogens, insecticides, or repellents. *A. aegypti* were reared following the protocols established in our laboratory by Gonzalez and Harburguer [28]. Briefly, eggs were laid on a wet filter paper and kept on the wet paper for 48 h. The eggs were dehydrated at ambient temperature and stored for at least 30 days. When needed, eggs were placed in dechlorinated water (500 eggs per 2 L of water), and after 24 h first instar larvae were observed. The larvae were fed a mixture of rabbit pellets and yeast. They were transferred to acrylic cages in the pupal instar and maintained in the same conditions with water and raisins until adults appeared. Non-blooded fed adult females between 3 and 5 days old were used.

**Fig. 1:**
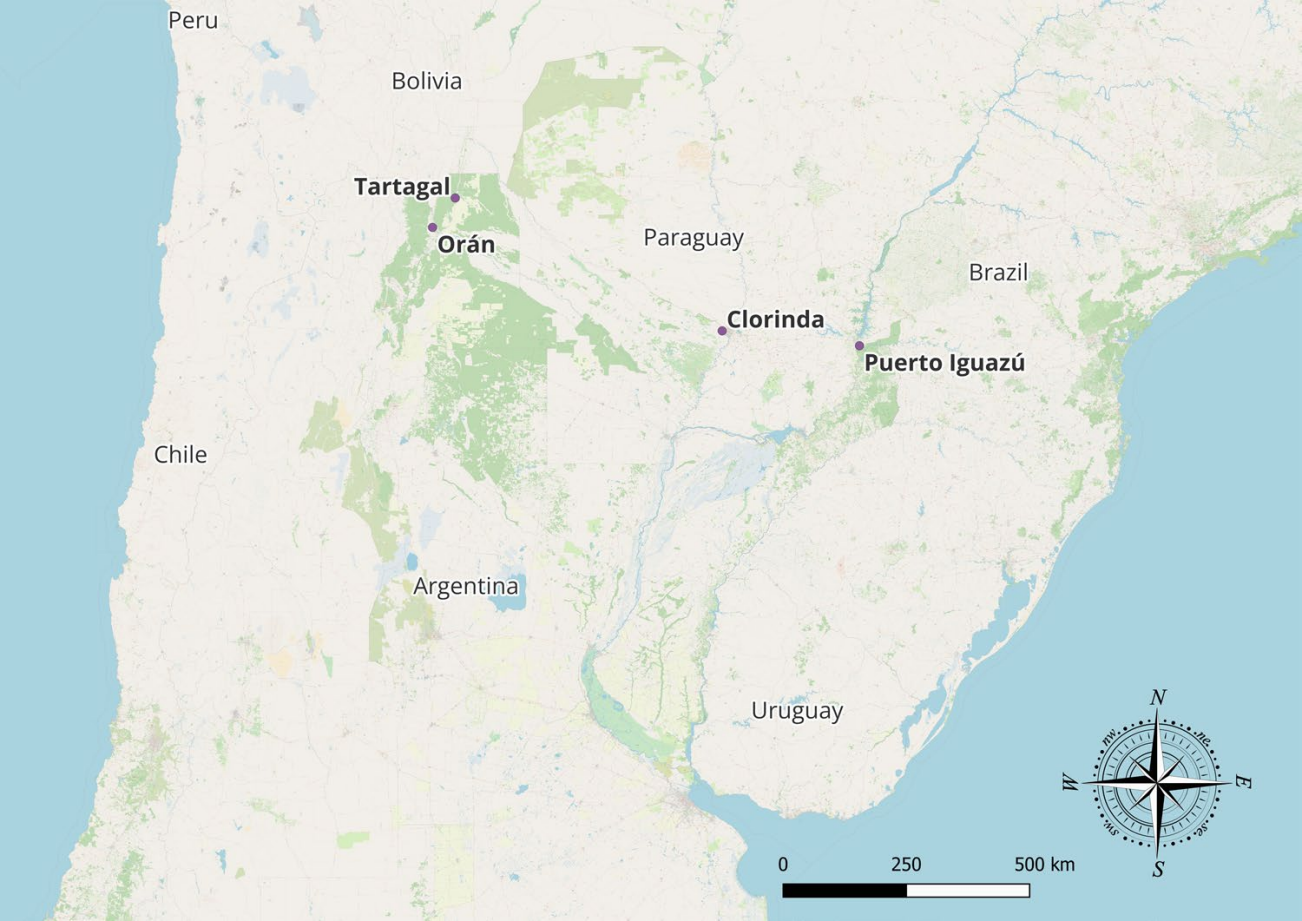
Location of cities where the *A. aegypti* eggs were collected. Tartagal and San Ramón de la Nueva Orán (Salta) close to Bolivia, Clorinda (Formosa) bordering Paraguay, and Puerto Iguazú (Misiones) bordering Paraguay and Brazil. Base map and data from OpenStreetMap and OpenStreetMap Foundation (CC-BY-SA) © https://www.openstreetmap.org and contributors. Edited with QGIS.org, 2024. QGIS Geographic Information System. QGIS Association

Ovitraps were used to collect *A. aegypti* eggs in the province of Salta (Argentina), in the cities of Tartagal and San Ramón de la Nueva Orán, which are located at 22°03’S 63°42’W and 23°08’S 64°19’W, respectively. Ovitraps were also used to collect eggs from Puerto Iguazú (Misiones) and Clorinda (Formosa), located at 25°36’S 54°34’W and 25°17’S 57°43’W (Fig. 1). These cities have a history of dengue cases and high *A. aegypti* entomological indices (Bulletin from the Ministry of Health Argentina [8]). Local entomology teams conducted ovitrap collections of *A. aegypti* eggs between September 2021 and April 2022. Ovitraps were prepared according to Seccacini et al. [29] using black plastic jars. The number of ovitraps installed was determined on the basis of the number of buildings, which serves as an indirect measure of the population density. For areas with 60,000 or fewer buildings, 100 ovitraps were installed [30]. The collected eggs were sent to Centro de Investigaciones de Plagas e Insecticidas (CIPEIN)’s laboratory and reared in the same manner as the susceptible strain. This study utilized 3- to 5-day-old adult females from the F1 generation.

### Insecticide-impregnated papers

Papers were impregnated according to WHO protocol [31], with the discriminating concentration (DC) of permethrin (0.4%), and the 5× and 10× (2% and 4%, respectively) using a mixture of silicone oil and acetone as carrier. Papers with pirimiphos methyl were impregnated at 60 mg/cm^2^ using only acetone as carrier. This active was chosen because it belongs to a chemical group different from pyrethroids and it is also recommended by the WHO for space spraying to control *A. aegypti*. We also impregnated papers with PBO at 4% as suggested by WHO, using a mixture of silicone oil and acetone as carrier. The papers were left to air dry overnight, then covered in aluminum foil, and kept at 4 °C until use.

The discriminating concentrations were chosen on the basis of the latest document published by the WHO that contains the results of a multicenter study to establish the new DC of the different insecticides used in public health [32].

Once impregnated, a sample of the permethrin papers were sent to Instituto de Ecologia y Desarrollo Sustentable (INEDES) to determine the amount of permethrin per cm^2^ by gas chromatography and to evaluate the homogeneity of the impregnation. The impregnated paper was systematically cut with a grid that contemplated a homogeneous sampling of six sectors. Each fraction of paper was placed in a vial with acetone to extract the insecticide. The acetone solution of permethrin was analyzed by gas chromatography coupled with mass spectrometry (GC–MS). A DB-5 nonpolar capillary column (30 m × 0.25 mm internal diameter and 0.25 μm thickness of the stationary phase; J&W Scientific, Folsom, CA, USA) was used. The column temperature was held at 60 °C for 1 min, then programmed to increase from 60 °C to 150 °C at a rate of 20 °C/min, held for 4 min, then increased to 290 °C at a speed of 15 °C/min. This final temperature was maintained for 17 min. The injector was set at 280 °C. Helium (99.99%) was used as the carrier gas at a flow rate of 1.2 mL/min. For the quantitative determination, selective ion monitoring (SIM method) was performed, and permethrin was identified through an ion of mass 183 (retention time: 20.15 min). The permethrin dose was determined for each sampled sector, expressed in milligrams of permethrin per cm^2^. From the data, it was possible to obtain a distribution map of the active ingredient in the total impregnated surface.

### Bioassays

The papers were placed into standard exposure tubes following WHO protocol [33]. The mosquitoes were kept in the exposure tubes for 1 h. After exposure, mosquitoes were transferred back to holding tubes, and a cotton pad soaked in 10% sugar water was placed on the mesh-screen end. The mosquitoes were kept in the holding tubes for 24 h (recovery period), and the number of dead mosquitoes was counted and recorded. The mortality percentages were adjusted using Abbott’s formula [34].

When PBO was used, before exposure to the insecticide, the female mosquitoes were exposed to this compound for 1 h, and then the same procedure described above was followed.

Between four and five replicates were carried out for each treatment (control, DC, 5×, 10×, DC + PBO, and pirimiphos-methyl) using between 18 and 25 mosquitoes per tube, making a total of between 72 and 125 mosquitoes evaluated per treatment. This makes a total of between 420 and 750 mosquitoes evaluated per population.

### *Kdr* genotyping

#### DNA extraction

DNA was individually extracted from individual mosquito females, with TNES lysis buffer and alcohol washes, following Martins et al. [35]. The pellet was resuspended in 30 µL ultra-pure water, quantified in a NanoDrop One (ThermoFisher), and diluted to 10 ng/µL in ultra-pure water.

#### Genotyping

We genotyped each sample for the three *kdr* single nucleotide polymorphisms (SNP), V410L, V1016I, and F1534C, in individual TaqMan qPCR reactions, with the same procedures, reagents, and PCR conditions described elsewhere [36]. Briefly, we used 20 ng DNA, 1× TaqMan Genotyping Master Mix (ThermoFischer), 1× TaqMan SNP Genotyping assay, and ultra-pure water q.s. 10 µL. The genotype of each separated SNP was analyzed with the online software Genotype Analysis Module V4.1 (Applied Biosystems, cloud platform). The genotype of each mosquito consisted in the combination of the three SNPs (410 + 1016 + 1534), which potentially resulted in 27 possible genotypes. On the basis of the observed genotypes, we presumed the phasing of each SNP variant to determine the *kdr* alleles and the genotypic and allelic frequencies (see Souza et al. [37]).

### Data analysis

Mortality in bioassays was calculated as the average mortality of 4–5 replicates. The resistance status of mosquitoes was determined using the WHO criteria [33]. When the DC was used; mortality ≤ 90% was considered as resistant, mortality ≥ 91–97% was suspected resistant, and mortality ≥ 98% was susceptible. When 5× concentration was applied, ≥ 98% mortality was considered as low-intensity resistance, and < 98% mortality was considered as moderate- to high-intensity resistance. When 10× concentration was applied, ≥ 98% mortality was considered as moderate-intensity resistance, and < 98% mortality was considered as high-intensity resistance.

Regarding the use of PBO, WHO guidelines state that a complete restoration of susceptibility implies that a monooxygenase-based resistance mechanism fully accounts for the expression of the resistant phenotype; partial restoration implies a monooxygenase-based resistance mechanism only partially explains resistance, while no restoration indicates non-monooxygenase-based resistance.

## Results

### Insecticide susceptibility assays

First, the quality of the paper impregnation was evaluated to ensure that it complied with the required standards. The theoretical concentration of permethrin (DC) in the paper should average 0.0146 mg/cm^2^ of active ingredient. The average value was quantified and its value did not differ significantly from the default theoretical value, yielding an average of 0.0141 ± 0.0011 mg/cm^2^. The confirmation of this value was extremely important to corroborate that the required amount of permethrin has been fully impregnated in the paper, not undergoing changes during its storage and transportation. This result also confirmed that the chemical extraction technique proposed by INEDES was adequate, since a total extraction of permethrin was obtained from the impregnated paper.

To study whether the impregnation was homogeneous, a maximum allowable coefficient of variation (CV%) of 10% was set. The analysis carried out by INEDES demonstrated that impregnated papers had a CV of 7.06%. Then, considering the threshold value of 10%, the papers were homogeneously impregnated.

**Fig. 2:**
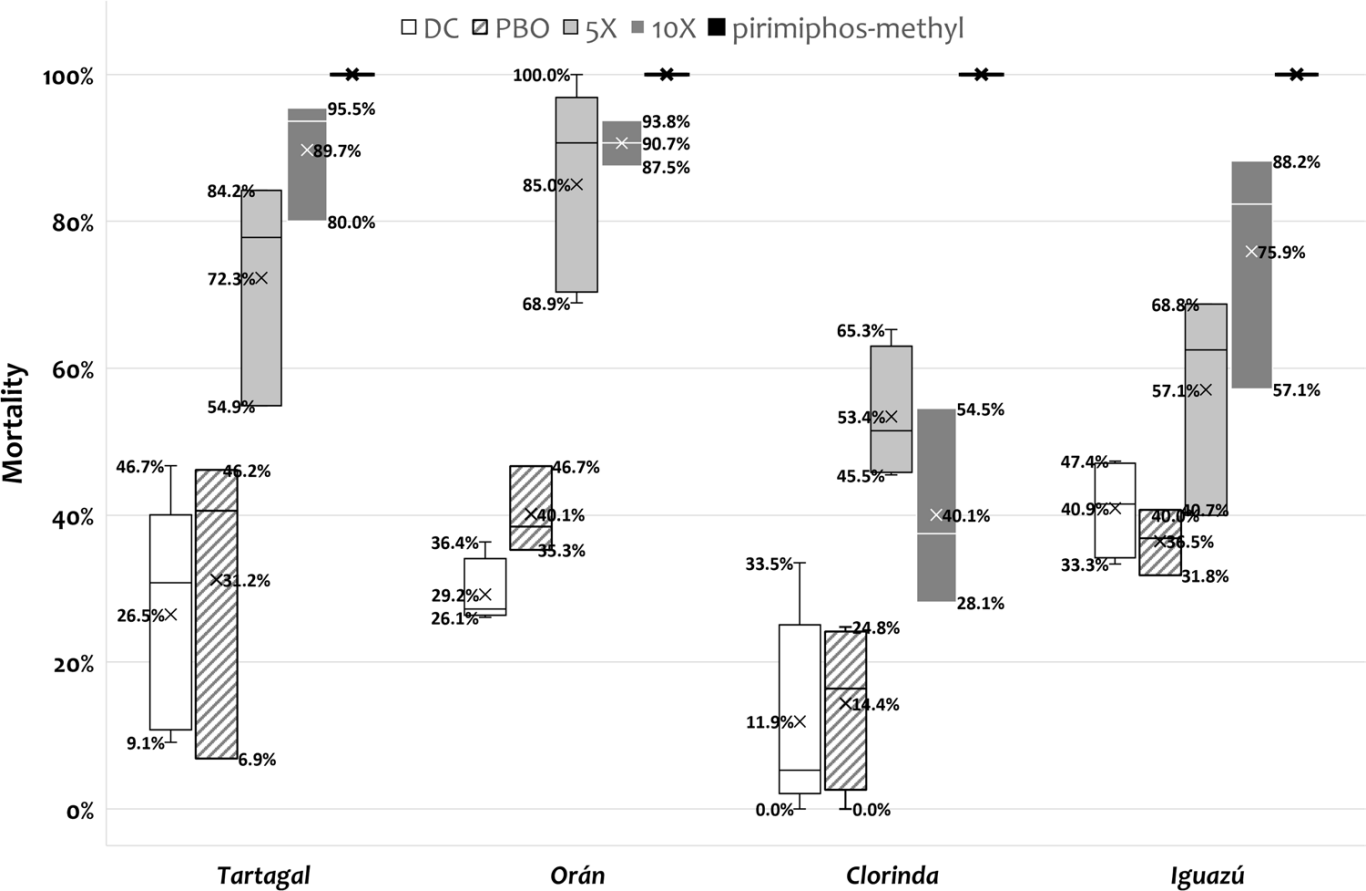
Mortality (%) of *Aedes aegypti* adults against insecticides for the different populations evaluated; x indicates the average mortality (%) for this treatment. For each treatment, the maximum and minimum mortality values obtained are also indicated. *DC* discriminating concentration, *PBO* treatment with piperonyl butoxide, *5×* five times the DC, *10×* ten times the DC

In the bioassays, the susceptible reference strain Rockefeller presented 100% mortality to the permethrin DC 0.4%. All the field populations showed a mean mortality below 90%, and were therefore considered resistant to permethrin: Tartagal (26.5%), Orán (29.2%), Puerto Iguazú (40.9%), and Clorinda (11.9%) (Fig. 2).

We then investigated the levels of resistance. The mean mortality percentage with the 5×DC (2%) permethrin remained below 98% in all populations: Tartagal (72.3%), Orán (85%), Clorinda (53.4%), and Iguazú (57.1%), and also with the 10×DC (4%): Tartagal (89.7%), Orán (90.7%), Clorinda (40.1%), and Iguazú (79.9%) (Fig. 2). These results indicated that all investigated *A. aegypti* populations were highly resistant to permethrin.

Exposition with PBO prior to the permethrin 0.4% increased mortality in the Orán mortality population by approximately 10% (from 29.2% to 40.1%). The synergism effect of PBO was not observed in the other populations, as the average mortality remained the same (as can be seen, the quartiles of the graphs overlap for all populations except Orán) (Fig. 2). These results suggested at least a partial participation of multifunction oxidases (P450) as a metabolic resistance mechanism in Orán population.

Finally, a control alternative for these populations based on the use of an organophosphate, pirimiphos-methyl, was evaluated. All populations were susceptible to the DC (60 mg/cm^2^) organophosphate, similarly to the reference control Rockfeller, all exhibiting 100% mortality (Fig. 2).

### *Kdr* genotyping

We obtained the genotypes of 160 *A. aegypti* mosquitoes for the three *kdr* SNPs; V410L, V1016I, and F1534C (Table 1). In this study, the presence of the V410L mutation was detected for the first time in Argentina. In total we found seven genotypes, all with at least one *kdr* SNP, i.e., the wild-type genotype VV + VV + FF was absent. The homozygous genotype *kdr* in the 1534 site only (VVC, aka *kdr* R1 allele) was not observed Iguazu, but in the other populations; however, in Clorinda, only one individual out of all those analyzed showed that genotype. The LL + II + CC genotype formed by the triple *kdr* allele (LIC, *kdr* R2) was present in all populations, at higher frequencies in Clorinda (83.3%) and Iguazu (55.6%). The northwestern populations Tartagal and Orán presented higher frequencies in the heterozygote genotype (VL + VI + CC, formed by the *kdr* alleles R1 and R2), with 50.0% and 37.5%, respectively. Further, three unusually observed genotypes were found, one in Tartagal (VV + VI + CC, 5%) and other two in Orán (VL + VV + CC, 2.4% and LL + VV + CC, 4.8%).

**Table 1:** Frequency of knock-down-resistant (*kdr*) alleles and genotypes found at Nav p.410, Nav p.1016, and Nav p.1534 positions in *Aedes aegypti* from Argentina

Populations	Genotypes (410 + 1016 + 1534) (*n*, %)									*N*_total_
	VV + VV + FF^1^	VV + VV + F**C**	VV + VV + **CC**	V**L** + V**I** + F**C**	V**L** + V**I** + C**C**	**LL** + **II** + **CC**	VV + V**I** + CC*	V**L** + VV + CC*	**LL** + VV + CC*	
Iguazú	0	0	0	0	16 (44.4%)	20 (55.6%)	0	0	0	36
Clorinda	0	0	1 (2.4%)	0	6 (14.3%)	35 (83.3%)	0	0	0	42
Tartagal	0	0	5 (12.5%)	4 (10%)	20 (50.0%)	9 (22.55%)	2 (5.0%)	0	0	40
Orán	0	0	11 (26.2%)	0	15 (35.7%)	13 (31.0%)	0	1 (2.4%)	2 (4.8%)	42

Alleles marked in **bold** capital letters correspond to the mutant genotype

^1^Corresponds to the wild-type genotype

^*^Unusually observed genotypes

## Discussion

The rapid spread of insecticide resistance poses a major challenge in mosquito control, potentially undermining the efficacy of current insecticide-based strategies. Unfortunately, there is a lack of accessible data on the prevalence and mechanisms of insecticide resistance in specific geographic locations. These data are crucial for guiding national programs in selecting the most effective compounds to contest resistance.

In this study, the susceptibility to permethrin of four *A. aegypti* field populations from Argentina was evaluated for the first time: Tartagal and Orán (Salta), Clorinda (Formosa), and Puerto Iguazú (Misiones). For those populations that were resistant using DC, a dose of 5× and 10× were evaluated to study the intensity of resistance. Likewise, exposure to PBO was carried out and the presence of *kdr* mutations was evaluated. Finally, alternatives for the control of resistant populations were studied.

All the populations studied were resistant to permethrin. According to the protocol established by the WHO, when the mortality of mosquitoes exposed to DC is less than 90%, the population should be considered resistant. In all the cases evaluated, mortality was much less than 90%.

PBO can enhance the effects of pyrethroid insecticides by reducing or neutralizing the detoxifying capabilities of enzymes, particularly monooxygenases. This suggests that if the monooxygenase-based detoxification system is mainly responsible for the resistance, a partial or complete reduction in the expression of the resistant phenotype can be observed. In the case of the populations we studied, when PBO was used, an increase in mortality was only observed in the Orán population, going from an average of 29.2% to 40.1%. As the mitigation of resistance is not complete, this could indicate that at least one of the mechanisms responsible for it is an increase in monooxygenases, but that there are possibly others. For the rest of the populations evaluated, no difference was observed with the use of PBO.

Furthermore, resistant phenotypes identified using the discriminating concentrations should be further evaluated for their potential operational significance. This can be done by exposing subsequent mosquito samples from the same target vector population to significantly higher concentrations of the relevant insecticides. Although these higher concentrations for each insecticide will not match their recommended field application rates, they will provide valuable information regarding the intensity of resistance.

For this reason, once the DC for permethrin had been evaluated, the intensity of the resistance was studied by subjecting all the populations to a dose five (5×) and ten (10×) times greater than the DC. Our results show that when all the populations were exposed to the 5×, mortality was less than 98%, indicating that these are populations with moderate to high resistance. When the populations were exposed to the 10×, mortality was also lower than 98%, indicating that all populations can be considered as highly resistant to permethrin. It is important to highlight the case of Clorinda, where the results using 5× and 10× are similar but mortality does not increase significantly by doubling the dose used, indicating that this population probably has a very high degree of resistance to permethrin. These results coincide with the only work carried out to date in Argentina that presents a toxicological evaluation, where high resistance to permethrin was found in Salvador Mazza (Salta) [13].

A control alternative for these populations based on the use of an organophosphate, pirimiphos-methyl, was evaluated. All the populations were susceptible to pirimifos-methyl, with 100% mortality for all replicates.

Single nucleotide changes in the voltage-gated sodium channel (NaV) are the predominant genetic alterations associated with insecticide resistance. These changes result in resistance to the knockdown effect of pyrethroids. Worldwide populations of *A. aegypti* have regularly shown substitutions in the 1016 and 1534 locations. While the wild-type Val is replaced by Ile (V1016I) and by Gly (V1016G) in American and Asian populations, respectively, the Phe to Cys substitution in the 1534 position (F1534C) is present in populations from the Americas, Africa, and Asia [39–41]. There have also been reports of other changes, such as the substitution of Val to Leu at position 410 (V410L) in populations from Latin America [25, 42]. It is known that the F1534C mutation in *A. aegypti* is linked to a low level of resistance to type I pyrethroids but not type II pyrethroids as well, and pyrethroid sensitivity was unaffected by V1016I alone [31, 32]. However, the V1016I genotype showed a greater reduction in susceptibility to type I and II pyrethroids than the F1534C mutant alone when both resistant genotypes are present simultaneously. Since then, F1534C and V1016I have both been found in numerous populations.

In this study the presence of the V410L mutation was detected for the first time in Argentina. Additionally, it has been observed in samples from Mexico since 2002 [38], and is currently highly disseminated [42]. The widespread presence of the V410L in all evaluated cities, strongly linked with V1016I (and therefore 1534Cys), the triple *kdr* allele, suggests a significant factor contributing to the high levels of resistance observed in all populations in this study, especially in the city of Clorinda, where the proportion of individuals that have the triple mutation is 83.3%, in accordance with the toxicological data where the lowest mortality (11.9% using the DC) is observed. In fact, as mentioned above, there are no differences in average mortality when individuals are exposed to 5× and 10×.

In addition, three unusually observed genotypes were found in Orán and Tartagal, both located in in Salta. In Yacuiva (Bolivia), a city close to Orán and Tartagal, resistance to deltamethrin in *A. aegypti* was detected [43]. The movement of people and goods between Yacuiba (Bolivia) and Salta (Argentina) is constant and intense. This may have led to the introduction of resistance genes from the Bolivian mosquito population to the Argentine strain [44]. In 1964, after a rigorous dichlorodiphenyltrichloroethane (DDT) control campaign led by the Pan American Health Organization (PAHO), *A. aegypti* was believed to have been completely eliminated from Argentina [45]. However, in 1986, there were reports of reinfestation in the northeastern provinces [46]. Genetic analysis has revealed distinct *A. aegypti* lineages in different regions of Argentina. Rondan Dueñas et al. [44] have identified three haplogroups that may represent different introductions of *A. aegypti* into South America from various origins. There is greater genetic variability in the populations of northwestern Argentina compared with those in the east and northeast. This diversity may indicate that the control campaign in the 1950s and 1960s, promoted by PAHO, was less effective in the northwest, allowing remnants of former colonizing populations to survive and rapidly increase in population density. It is also possible that the original mosquito populations were eliminated from the east and northeast regions and subsequently recolonized by a few new haplotypes. Furthermore, Albrieu Llinas and Gardenal [47] showed that haplotypes from the northwestern region of Argentina and Bolivia were clustered together in the same clade, while those from the northeastern region, Brazil, and Paraguay were clustered in an opposite extreme [48]. These two studies may partially explain why this unusual combination of *kdr* genotypes appears only in the two cities located further northwest.

Valle et al. [49] conducted a systematic review of the resistance statuses of Brazilian *A. aegypti* populations from 1985 to 2017 against deltamethrin, the primary compound used nationally to control *A. aegypti* adults. The review revealed widespread resistance to this pyrethroid throughout the country, including the state of Paraná, which borders Misiones, Argentina. Because of these findings, the use of pyrethroids in Paraná was discontinued in 2007 and replaced with the organophosphate malathion. Consequently, it is possible that Puerto Iguazú (Misiones) populations could share pyrethroid resistance genes with Foz do Iguazú populations in the state of Parana, Brazil, although no studies have confirmed this assumption.

The extremely high levels of pyrethroid resistance caused by the V410L mutation, either alone or in combination with F1534C, could render pyrethroids largely ineffective in Argentine regions where *A. aegypti* mosquitoes have these mutations. Additionally, this situation could exacerbate the problem by increasing the selection pressure for resistant individuals. The findings have significant implications for decision-makers involved in vector control programs in Argentina within the framework of integrated vector management (IVM). They indicate an urgent necessity to introduce a new insecticide with a different mode of action in these regions.

## Conclusions

This study demonstrates for the first time the presence and intensity of resistance to permethrin in the populations of Tartagal and San Ramón de la Nueva Orán (Salta), Clorinda (Formosa), and Puerto Iguazú (Misiones) in Argentina, and correlates the observed phenotype with the presence of *kdr* mutations (genotype). In addition, pirimifos-methyl is proposed as a control alternative since a 100% mortality was obtained for all the populations evaluated.

## Data Availability

No datasets were generated or analyzed during the current study.
